# Genetic population structure of endangered ring‐tailed lemurs (*Lemur catta*) from nine sites in southern Madagascar

**DOI:** 10.1002/ece3.6337

**Published:** 2020-07-16

**Authors:** Aparna Chandrashekar, Jessica A. Knierim, Sohail Khan, Dominique L. Raboin, Sateesh Venkatesh, Tara A. Clarke, Frank P. Cuozzo, Marni LaFleur, Richard R. Lawler, Joyce A. Parga, Hantanirina R. Rasamimanana, Kim E. Reuter, Michelle L. Sauther, Andrea L. Baden

**Affiliations:** ^1^ Department of Anthropology Hunter College of the City University of New York New York NY USA; ^2^ Animal Behavior and Conservation Program Department of Psychology Hunter College of the City University of New York New York NY USA; ^3^ Department of Sociology and Anthropology North Carolina State University Raleigh NC USA; ^4^ Lajuma Research Centre Louis Trichardt (Makhado) South Africa; ^5^ Department of Anthropology University of San Diego San Diego CA USA; ^6^ Lemur Love, Inc. San Diego CA USA; ^7^ Department of Sociology and Anthropology James Madison University Harrisonburg VA USA; ^8^ Department of Anthropology California State University‐Los Angeles Los Angeles CA USA; ^9^ Ecole Normale Supérieure University of Antananarivo Antananarivo Madagascar; ^10^ Pet Lemur Survey Housed by the University of Utah Salt Lake City UT USA; ^11^ Department of Anthropology University of Colorado Boulder Boulder CO USA; ^12^ Department of Anthropology The Graduate Center of the City University of New York New York NY USA; ^13^ The New York Consortium in Evolutionary Primatology (NYCEP) New York NY USA; ^14^ Present address: San Diego Zoo Global Institute for Conservation Research San Diego CA USA; ^15^ Present address: Wildlife Alliance New York NY USA; ^16^ Present address: Rutgers University New Brunswick NJ USA

**Keywords:** conservation genetics, Madagascar, microsatellites, strepsirrhines

## Abstract

Madagascar's ring‐tailed lemurs (*Lemur catta*) are experiencing rapid population declines due to ongoing habitat loss and fragmentation, as well as increasing exploitation for bushmeat and the illegal pet trade. Despite being the focus of extensive and ongoing behavioral studies, there is comparatively little known about the genetic population structuring of the species. Here, we present the most comprehensive population genetic analysis of ring‐tailed lemurs to date from across their likely remaining geographic range. We assessed levels of genetic diversity and population genetic structure using multilocus genotypes for 106 adult individuals from nine geographically representative localities. Population structure and *F*_ST_ analyses revealed moderate genetic differentiation with localities being geographically partitioned into northern, southern, western and also potentially central clusters. Overall genetic diversity, in terms of allelic richness and observed heterozygosity, was high in the species (AR = 4.74, *H*_O_ = 0.811). In fact, it is the highest among all published lemur estimates to date. While these results are encouraging, ring‐tailed lemurs are currently affected by ongoing habitat fragmentation and occur at lower densities in poorer quality habitats. The effects of continued isolation and fragmentation, coupled with climate‐driven environmental instability, will therefore likely impede the long‐term viability of the species.

## Graphical abstract

Here, we present the most comprehensive population genetic analysis of ring‐tailed lemurs to date from across their likely remaining geographic range. Our study uses multilocus genotypes for 106 adult individuals from nine geographically representative localities to evaluate population genetic diversity and structure on a range‐wide scale. The results described herein demonstrate that, while Madagascar's remaining ring‐tailed lemur populations are geographically isolated, they have retained high levels of genetic diversity, with moderate genetic differentiation among populations despite a relatively large geographic distribution.

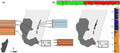

## 1 INTRODUCTION

Sustaining natural levels of genetic diversity within wildlife populations is a key concern for conservation biologists (Frankham, 1995, 2003, 2005). Pressures from climate change, anthropogenic habitat modification, overexploitation, and the introduction of novel competitors and infectious diseases are producing rapidly and ever‐changing environments, forcing species to adapt and evolve or go extinct (Di Marco, Venter, Possingham, & Watson, 2018; Frankham, Ballou, & Briscoe, 2010). Genetic diversity, the variation of alleles and genotypes present within a population, is the foundation on which natural selection acts and is therefore necessary for adaptive evolutionary change to occur (Frankham, 1995, 2003, 2005; Frankham et al., 2010). Populations with low levels of genetic diversity struggle to evolve in modified environments. For instance, the Tasmanian devil (*Sarcophilus harrisii*) has become vulnerable to the spread of devil facial tumor disease due to the lack of diversity across immune genes following human‐induced population crashes from introduced diseases (Guiler, 1970; Morris, Wright, Grueber, Hogg, & Belov, 2015).

Habitats modified by human activity hold less genetic diversity than pristine environments, thus putting their inhabitants at high risk (Miraldo et al., 2016). That is because deforestation, fragmentation, and habitat degradation—all critical threats to biodiversity—interact to restrict the amount of viable habitat available to species, reduce carrying capacity and consequently maximum population size, and create isolated patches separated by matrix (i.e., inhospitable habitat) that impedes gene flow among remaining species' populations (Baden et al., 2019; Holmes et al., 2013; Stangel, Lennartz, & Smith, 1992). Combined, these processes lead to greater inbreeding, reduced genetic diversity, and ultimately an increased extinction risk (Frankham et al., 2010; Lino, Fonseca, Rojas, Fischer, & Pereira, 2019; Struebig et al., 2011).

Though not alone in its vulnerability to habitat loss, Madagascar's biodiversity is considered to be a top concern, in part because of its incredible concentration of species endemism (Myers, Mittermeier, Mittermeier, da Fonseca, & Kent, 2000). Since its colonization by humans as recently as 4,000 years ago, the island has undergone extensive forest cover loss and with it more than 17 species of large‐bodied lemurs (Dewar et al., 2013; Godfrey & Irwin, 2007; Kistler et al., 2015; Myers et al., 2000). Unfortunately, deforestation in Madagascar continues unabated (Vieilledent et al., 2018) and scientists anticipate that remaining rainforest habitat will be lost before the end of this century (Morelli et al., 2020). When considered alongside the impacts of climate change, this threat poses significant risk to the persistence of remaining extant lemur species (Brown & Yoder, 2015; Morelli et al., 2020). It is therefore an urgent conservation priority to quantify the genetic variability present within Madagascar's only endemic primate radiation to assess whether and to what extent lemur species can cope with intensifying environmental pressures.

**FIGURE 1:**
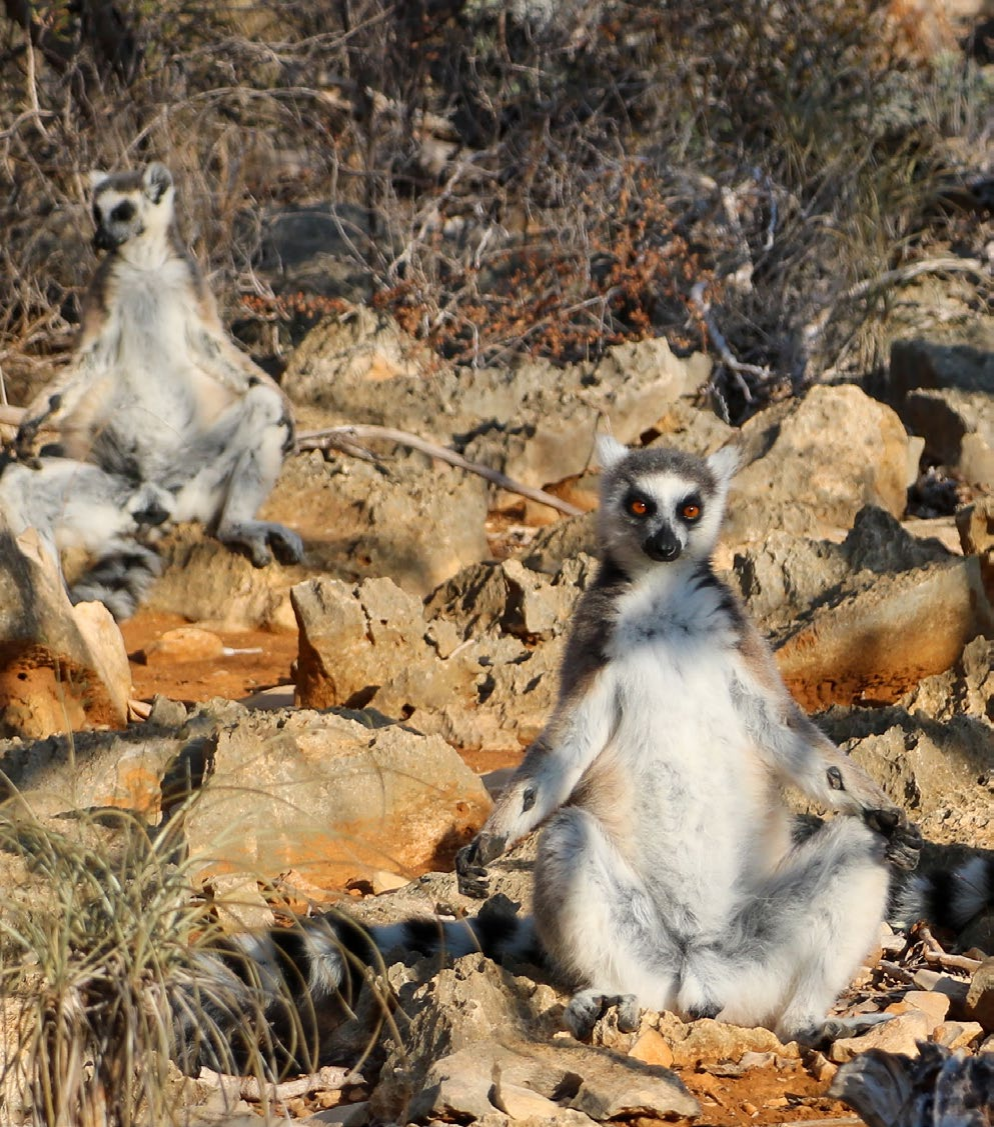
Photograph of ring‐tailed lemur (*Lemur catta*) by M LaFleur

Of particular concern is Madagascar's most charismatic species, the ring‐tailed lemur (*Lemur catta*, Figure 1). Ring‐tailed lemurs are medium‐sized (average 2.2 kg) terrestrial strepsirrhines that can be found throughout southern Madagascar (Cameron & Gould, 2013; Fardi, Sauther, Cuozzo, Youssouf, & Bernstein, 2018; Gould, Sussman, & Sauther, 2003; Sauther, Gould, Cuozzo, O'Mara, 2015; Sussman, 1991). They are considered a generalist taxon, maintaining a diverse frugivorous–folivorous diet (Cameron & Gould, 2013; Sauther, 1998; Sauther, Sussman, & Gould, 1999) and exhibiting considerable ecological flexibility (Cameron & Gould, 2013; Fardi et al., 2018; Gould et al., 2003; Sussman, 1991). The species occupies diverse habitat types ranging from rainforests to subalpine, deciduous, gallery, and spiny bush forests to anthropogenic savannah (Cameron & Gould, 2013; Gabriel, 2013; Goodman & Langrand, 1996; Goodman et al., 2002; Goodman, Rakotoarisoa, & Wilme, 2006; LaFleur & Gould, 2009; Sauther et al., 2006); however, much of their habitat has been altered by human activities, including clearing for agriculture, burning for charcoal production, and deforesting areas to create settlements (Sussman, Green, Porton, Andrianasolondraibe, & Ratsirarson, 2003). In the past 40 years alone, ring‐tailed lemurs have lost over 45% of their habitat (Brinkmann, Noromiarilanto, Ratovonamana, & Buerkert, 2014; LaFleur, Clarke, Ratzimbazafy, & Reuter, 2017a); and by 2080, it is estimated that 63% of their remaining range will shift due to climate change alone (Brown & Yoder, 2015). Furthermore, there has been a recent uptick in exploitation for the illegal pet trade, causing severe population declines, and in some cases local extinctions, throughout their remaining geographic range (Gardner & Davies, 2014; Gould & Sauther, 2016; LaFleur, Clarke, Reuter, Schaefer, & terHorst, 2019; LaFleur, Clarke, Reuter, & Schaeffer, 2017b; LaFleur & Gould, 2009; Reuter et al., 2019; Reuter & Schaefer, 2016). At present, there are estimated to be fewer than 2,400 individuals within sampled locations (Gould & Sauther, 2016; LaFleur et al., 2017b), though population estimates are still lacking throughout much of their range (e.g., Murphy, Ferguson, & Gardner, 2017).

Despite being one of the most‐studied lemur species, there is relatively little known about the genetic diversity and population structure of remaining wild ring‐tailed lemur individuals. Existing studies suggest that northern (Anja Community Reserve, Sakaviro, and Tsaranoro Valley Fragments; Clarke, Gray, Gould, & Burrell, 2015) and western localities (Bezà Mahafaly Special Reserve and Tsimanampetsotsa National Park; Parga, Sauther, Cuozzo, Jacky, & Lawler, 2012) maintain moderate levels of genetic diversity, with the smallest fragments (e.g., Sakaviro), isolated by roads and anthropogenic savannah, containing relatively lower levels of allelic richness and “mean number of alleles” than the larger western localities (Clarke et al., 2015; Parga et al., 2012). Moreover, low *F*_ST_ values among sites in the north (Clarke et al., 2015) and among those in the west (Parga et al., 2015) indicate minimal genetic differentiation, suggesting the presence of historical gene flow. While encouraging, there is also evidence that genetic erosion within the species has already begun to negatively impact their health and fitness (e.g., Charpentier, Williams, & Drea, 2008; Grogan, Sauther, Cuozzo, & Drea, 2017). It is therefore likely that at least some of Madagascar's remaining ring‐tailed lemur populations are already experiencing a time‐delayed response (i.e., extinction debt), as extinctions do not typically occur until several generations after a fragmentation event (Jackson & Sax, 2010; Tilman, May, Lehman, & Nowak, 1994).

In an effort to characterize the remaining genetic diversity present within the species and identify how this diversity is apportioned among remnant populations, we provide a preliminary population genetic assessment of ring‐tailed lemurs across their remaining geographic range. We evaluate within‐ and across‐site levels of genetic diversity and infer population genetic structure to better understand this species' adaptive potential and diagnose possible conservation priorities.

## 2 METHODS

### 2.1 Sample collection

**FIGURE 2:**
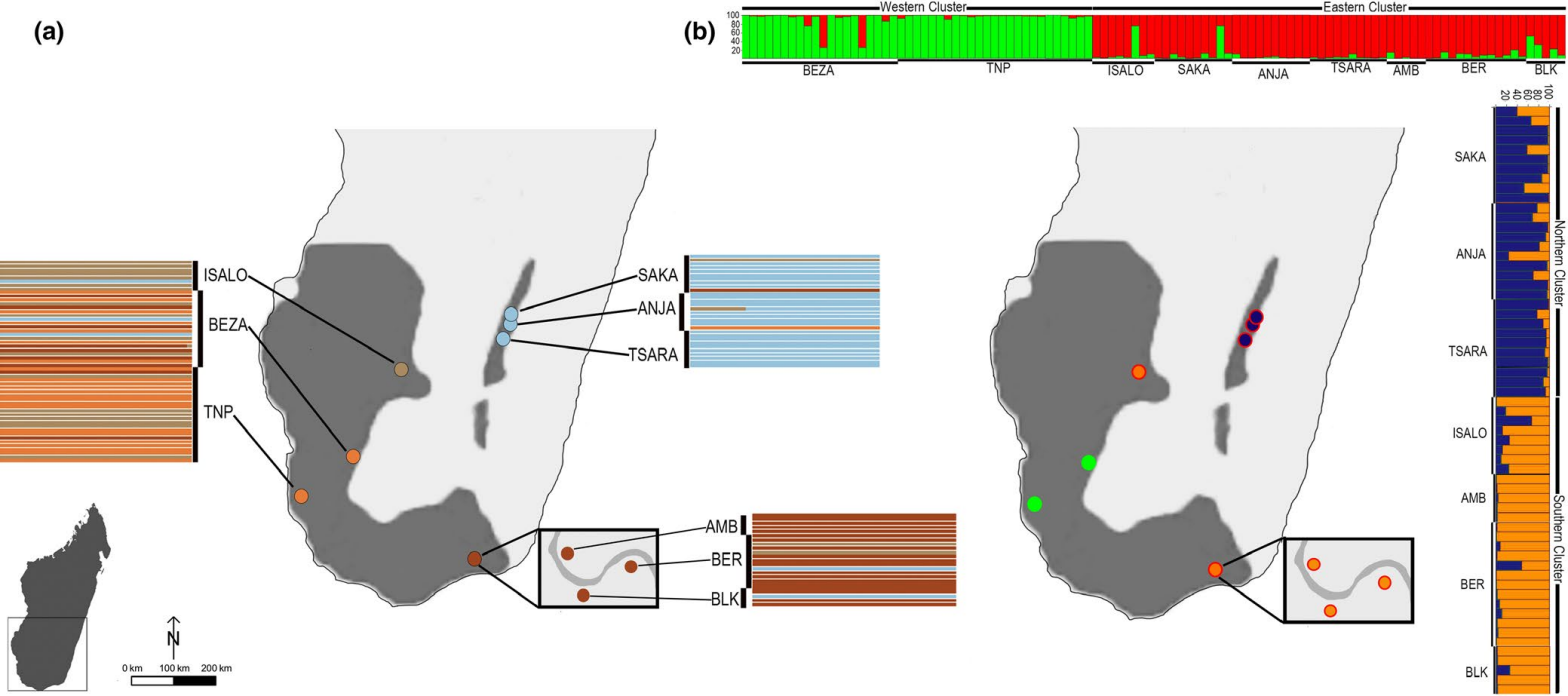
Map illustrating the nine ring‐tailed lemur localities sampled and population genetic structure results from DAPC (a) and Structure (b) analyses. Each bar illustrates the proportional membership (Q) of each individual lemur belonging to the clusters identified. DAPC (a) identified four genetic clusters (orange: west; beige: central; blue: north; brown: south). Structure (b) identified three genetic clusters. The horizontal bar (b) illustrates results from primary Structure analysis (*n* = 9 sites), showing that localities partitioned into eastern and western genetic clusters, as indicated by red and green point outlines, respectively. Vertical bar (b) illustrates results from secondary Structure analysis of eastern sites (*n* = 7 sites), indicating further subdivision into northern and southern clusters as indicated by blue and orange point fills, respectively. For full site names see Table 1. Gray shading illustrates historic ring‐tailed lemur distribution across southern Madagascar retrieved from IUCN website

Our sample included 106 adult ring‐tailed lemurs from nine geographically representative localities from across their existing range (Table 1, Figure 2). This dataset includes previously published genetic data from 75 adult ring‐tailed lemurs from five localities (Clarke et al., 2015; Parga et al., 2012), as well as 31 new individuals from an additional four sites (Table 1). Published genetic data were collected in May through August 2006 (Parga et al., 2012) and August to October 2012 (Clarke et al., 2015). Data for new individuals were generated from fecal samples collected from Isalo in July 2016 and from Ambirary (AMB), Beoloke (BLK), and Berenty (BER) in June and July 2017. Multiple individuals and groups were sampled at each locality (Table 1). Fecal samples were immediately stored in RNA*later* (Ambion)  to prevent DNA degradation and were banked within 1 month of collection at −80°C for long‐term storage. Sample collection and export/import protocols adhered to Malagasy and International laws and were approved by Malagasy wildlife authorities and the US Fish and Wildlife Service.

**TABLE 1:** Sampling localities, geographic coordinates, and sample sizes of ring‐tailed lemur populations used in this study

Site name	Site code	Geographic region	Habitat type	Protection status	Latitude	Longitude	*n* ind.	*n* groups	Reference
Ambirary P.R.	AMB	SE	Gallery, scrub, and spiny forest	Private Reserve	24°59′53″ S	46°18′07″ E	5	3	This study
Anja C.R.	ANJA	NE	Mixed xerophytic, deciduous vegetation, large granite outcrops	Community‐level association	21°51′12″ S	46°50′40″ E	10	3	Clarke et al. (2015)
Beoloke P.R.	BLK	SE	Gallery forest	Private Reserve	25°00′59″ S	46°18′51″ E	5	4	This study
Berenty P.R.	BER	SE	Gallery, scrub, and spiny forest	Private Reserve	25°00′00″ S	46°18′00″ E	13	8	This study
Beza Mahafaly S.R.	BEZA	W	Gallery forest	Special Reserve	23°30′00″ S	44°40′00″ E	20	6	Parga et al. (2012)
Isalo N.P.	ISALO	C	Grass savannah, large rock formations	National Park	22°29′26″ S	45°22′44″ E	8	2	This study
Sakaviro	SAKA	NE	Mixed xerophytic, deciduous vegetation, large granite outcrops	Community‐level association	21°47′03″ S	46°52′02″ E	10	During census	Clarke et al. (2015)
Tsaranoro V.F.	TSARA	NE	Mixed xerophytic, deciduous vegetation, large granite outcrops	Community‐level association	22°05′11″ S	46°46′14″ E	10	4	Clarke et al. (2015)
Tsimanampetsotsa N.P.	TNP	W	Spiny, succulent, xerophilic vegetation	National Park	24°06′00″ S	43°50′00″ E	25	4	Parga et al. (2012)
Total sample							106	34	

Abbreviations: C, central; CR, Community Reserve; NE, northeast; NP, National Park; PR, Private Reserve; SE, southeast; SR, Special Reserve; VF, Valley Forest; W, west.

### 2.2 DNA extraction

Total genomic DNA (gDNA) was extracted from new samples (*n* = 31) using QIAamp DNA Stool Mini Kits (QIAGEN) following Clarke et al. (2015). Samples were amplified at six microsatellite markers that have been shown to reliably amplify fecal DNA: L‐2 (Merenlender, 1993), Lc5, Lc6, Lc7 (Pastorini, Fernando, Forstner, & Melnick, 2005), 69HDZ267, and 69HDZ299 (Zaonarivelo et al., 2007) (Appendix S1).

### 2.3 Microsatellite genotyping

Extraction products were amplified via PCR in a 13 μl reaction volume using 6.25 μl HotStarTaq DNA polymerase Master Mix, 20 mg/ml BSA, 10 μM primer pairs, and 3 μl (0.25–1 ng) gDNA using annealing temperatures outlined in the Appendix S1. The 5′ end of the forward primer was fluorescently labeled. PCR products were separated by capillary electrophoresis (ABI 3730xl Genetic Analyzer), and alleles were sized to an internal size standard (Rox‐500) using GeneMarker software v.2.6.7 (SoftGenetics). Genotype assignment was based on multiple independent reactions, where heterozygotes were confirmed with at least two independent reactions and homozygotes were confirmed with five independent reactions (Morin, Chambers, Boesch, &amp; Vigilant, 2001; Taberlet et al., 1996). Individuals from earlier studies (*n* = 45, Parga et al., 2012; *n* = 30, Clarke et al., 2015) were regenotyped and scaled to ensure datasets were comparable. CERVUS v.3.0 (Kalinowski, Taper, & Marshall, 2007) was used to calculate probability of identity (*P*_ID_), that is, the probability that two randomly drawn individuals from a population will show identical multilocus genotypes.

### 2.4 Population genetic analysis

#### 2.4.1 Genetic diversity

Using Micro‐Checker (van Oosterhout, Hutchinson, Wills, & Shipley, 2004), loci were checked for the presence of null alleles and were tested for deviations from Hardy–Weinberg equilibrium and linkage disequilibrium using the program Genepop v.4.2 (Raymond & Rousset, 1995). They were evaluated using a 10,000 iteration dememorization phase, followed by 100 batches of 10,000 iterations. Measures of genetic diversity, including number of alleles per locus (nA), mean number of alleles per locus (MNA), allelic richness (AR), observed (*H*_O_), and expected (*H*_E_) heterozygosities, and Wright's *F*_IS_ for each sampling location were calculated using GenoDive (Meirmans & Van Tienderen, 2004). We standardized allelic richness (AR) to the smallest sample size in the dataset to account for uneven sampling between populations using HP‐Rare 1.1 (Kalinowski, 2005).

#### 2.4.2 Population genetic structure

To assess the genetic distances between sampling localities, Wright's *F*_ST_ (Weir & Cockerham, 1984) was calculated for all pairs of populations using GenoDive (Meirmans & Van Tienderen, 2004). *F*_ST_ is a measure of genetic differentiation among subpopulations and illustrates whether and to what extent populations are considered genetically distinct (Frankham et al., 2010). Significance was calculated using 10,000 permutations corrected for multiple comparisons (Bonferroni adjusted *p* = 0.001).

The presence of isolation‐by‐distance (IBD) was evaluated using the program GenAlEx v.6.5 (Peakall & Smouse, 2012) and significance estimated with Mantel's test using 10,000 permutations (Mantel, 1967). Genetic distances between populations were estimated using *F*_ST_/(1 − *F*_ST_).

To identify genetic clusters, we used three different methods. First, we used a model‐based Bayesian clustering method implemented in Structure v2.3.4 (Pritchard, Stephens, & Donnelly, 2000). This method is used to estimate the number of genetically distinct clusters (*K*) with no a priori information regarding the individuals' geographic sampling locations provided, so clusters were formed solely on genetic information. We evaluated the hypothesis *K* = 1–12, three more than the number of wild populations (Evanno, Regnaut, & Goudet, 2005), using 100,000 iterations of MCMC following a burn‐in of 50,000 iterations, as longer burn‐in or MCMC did not significantly change our results. We implemented 20 runs for each value of *K* and assumed admixture and correlated allele frequencies. The admixture model allows individuals to have mixed ancestry, assuming that a portion of an individual's genome, *q*, comes from a subpopulation*, k* (where $\sum_{k}q_{k}=1$ ; Falush, Stephens, & Pritchard, 2003). To account for unbalanced sampling, the ALPHA value was changed from the default value (1.0) to 0.5 (Wang, 2016). The most likely number of genetic clusters (*K*) was assessed using the highest value of ∆*K* (Evanno et al., 2005) using the program Structure Harvester v0.6.94 (Earl & vonHoldt, 2012). Structure Harvester calculates the second‐order rate of change of the likelihood distribution (Δ*K*), which indicates the most pronounced subdivision within the data and the optimal number of genetic clusters. We implemented a two‐step approach to evaluate further substructure in the dataset. We first identified the most likely number of clusters within the overall sample (*n* = 106) and then ran subsequent analyses within each of the *K* clusters (Evanno et al., 2005).

To corroborate the Structure analysis, a discriminant analysis of principal components (DAPC) was performed in R, using the *adegenet* package (Jombart, 2008; R Core Team, 2017). This multivariate method identifies clusters of genetically related individuals that maximize between‐group variability and minimize within‐group variability by using a set of retained principal components (determined by the user to optimize variance explained; Jombart, Devillard, & Balloux, 2010). The optimal number of clusters is determined by the number of clusters with the smallest Bayesian information criterion (BIC) value.

To further substantiate both the Structure and DAPC results, a principal coordinates analysis (PCoA) was performed with a standard genetic distance matrix (Nei, 1978) using GenAlEx v.6.5 (Peakall & Smouse, 2012).

## 3 RESULTS

### 3.1 Genetic diversity

The nonexclusion combined probability of identity (*P*_ID_; Paetkau & Strobeck, 1994) of the six markers used in this study was 5.22 × 10^−10^ and for *P*_ID‐sibs_ was 1.13 × 10^−3^. These values demonstrate a low probability that two individuals would share the same multilocus genotype. The six loci were highly polymorphic, with the number of alleles ranging between 12 and 15 alleles (Table 2). Individuals were pooled across sampling localities, and there was no evidence of linkage disequilibrium. One locus was found to significantly deviate from Hardy–Weinberg equilibrium (L‐2; Table 2); however, it did not deviate at any one site specifically and was therefore included in further analyses.

**TABLE 2:** Characteristics of 6 microsatellite markers amplified in 106 ring‐tailed lemur samples, including the number of alleles per locus (*n*_A_), observed (*H*_o_) and expected (*H*_e_) heterozygosity, deviations from Hardy–Weinberg equilibrium (HWE), and polymorphic information content (PIC)

Marker	Size range (bp)	Annealing temp (°C)	*n*_A_	*H*_o_	*H*_e_	HWE	PIC	GenBank
L‐2	179–203	48	15	0.812	0.881	**0.0294**	0.853	–
Lc5	127–151	60	12	0.680	0.847	0.5353	0.835	AY366441
Lc6	248–270	60	12	0.788	0.809	0.3804	0.791	AY366442
Lc7	172–198	60	14	0.805	0.862	0.2387	0.857	AY366443
69HDZ267	156–178	55	15	0.902	0.916	0.1641	0.901	EF093488
69HDZ299	238–262	58	15	0.881	0.915	0.8349	0.896	EF093489

Note

Significant *p* values (*p* < 0.05) are shown in bold.

As a species, ring‐tailed lemurs maintain high levels of genetic diversity despite severe habitat fragmentation across their range. Mean number of alleles (MNA) ranged from 4.33 to 8.67 (Table 3). The mean observed heterozygosity across sampling sites was 0.811 ± 0.044, while mean expected heterozygosity was 0.775 ± 0.054. Overall *F*_IS_ was −0.052, and values ranged from −0.194 at ISALO to 0.042 at BER (Table 3).

**TABLE 3:** Allelic diversity within each of the nine ring‐tailed lemur sampling localities, including mean number of alleles (MNA), allelic richness (AR), observed (*H*_o_) and expected (*H*_e_) heterozygosity, inbreeding coefficient (*F*_IS_), and significant deviations from Hardy–Weinberg Equilibrium (HWE) calculated using 10,000 iterations

Site	*N*	MNA	AR(*SE*)	*H*_o_	*H*_e_	*F*_IS_	HWE
AMB	5	4.667	4.67	0.833	0.779	−0.075	0.7111
BLK	5	4.333	4.33	0.836	0.803	−0.042	0.9666
BER	13	8.000	5.88	0.828	0.865	0.042	0.2995
ANJ	10	4.833	4.19	0.771	0.716	−0.077	0.3493
SAKA	10	4.667	4.09	0.857	0.727	−0.179	**0.0039**
TSARA	10	6.167	4.91	0.722	0.730	0.011	0.7723
ISALO	8	4.667	4.19	0.854	0.719	−0.194	0.1452
BEZA	20	8.500	5.15	0.792	0.821	0.035	0.3109
TNP	25	8.667	5.23	0.807	0.816	0.011	0.2090
Overall	106	6.056	4.74	0.811	0.775	−0.052	‐

Note

Significant values (*p* < 0.05) are shown in bold.

### 3.2 Population genetic structure

**FIGURE 3:**
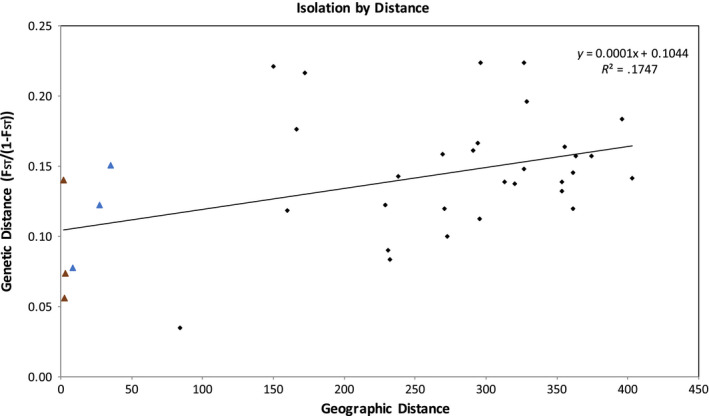
Results from Mantel's test for isolation‐by‐distance (IBD) in ring‐tailed lemurs from the nine localities sampled. The closest geographic localities (AMB‐BER‐BLK, brown triangles; and SAKA‐ANJA‐TSARA, blue triangles) did not reflect IBD

Pairwise values of *F*_ST_ among sampling localities ranged from 0.034 to 0.183, with a mean of 0.129. Genetic differentiation among sampling localities was significant in 31 out of 36 cases with nonsignificant *F*_ST_ comparisons between eastern localities AMB‐BLK‐BER, AMB‐ANJA, and ANJA‐SAKA (Table 4). Wright (1978), perhaps somewhat subjectively, considered *F*_ST_ values between 0.05 and 0.15 to indicate moderate genetic differentiation, whereas values of 0.15–0.25 to indicate great genetic differentiation (though Wright's recommendations were made at a time when highly mutable genetic markers, such as microsatellites, were not used). Pairwise distances (*F*_ST_) between BER and BLK (0.053), BER and AMB (0.071), and between BEZA and TNP (0.034) indicate minimal to moderate genetic differentiation between these localities and are consistent with the clustering seen in the subsequent Structure analyses. Larger values of *F*_ST_ (>0.15), suggesting great differentiation, were observed between ISALO and sampling locales in the north (SAKA and TSARA) and south (AMB and BLK). Despite great geographic distance, there was only moderate *F*_ST_ between BEZA and BER (0.077), BEZA and BLK (0.083), and TNP and BER (0.091). The results of Mantel's test (Figure 3) revealed a significant pattern of isolation‐by‐distance (IBD; *R* = 0.418, *p* = 0.007 based on 1,000 permutations) with geographical distance explaining over 17% of the variation in genetic distance. However, there was a considerable amount of unexplained variation in the IBD data. For example, in the eastern cluster there is relatively little geographic distance between the three populations comprising the northern (ANJA‐SAKA‐TSARA) and southern groups (AMB‐BER‐BLK), yet some sampling sites within each of these triads reflect moderate genetic differentiation (Figure 3).

**TABLE 4:** Pairwise *F*_ST_ values (above diagonal) and indication of significant *F*_ST_ values (below diagonal) among sampling localities of ring‐tailed lemurs

	AMB	BLK	BER	ANJA	SAKA	TSARA	ISALO	BEZA	TNP
AMB	–	0.125	0.071	0.124	0.129	0.185	0.146	0.111	0.139
BLK	NS	–	0.053	0.141	0.136	0.164	0.183	0.083	0.107
BER	NS	NS	–	0.117	0.107	0.129	0.101	0.077	0.091
ANJA	NS	*	*	–	0.072	0.109	0.150	0.122	0.155
SAKA	*	*	*	NS	–	0.131	0.178	0.121	0.124
TSARA	*	*	*	*	*	–	0.181	0.139	0.136
ISALO	*	*	*	*	*	*	–	0.106	0.125
BEZA	*	*	*	*	*	*	*	–	0.034
TNP	*	*	*	*	*	*	*	*	–

Note

Significant values indicated with * (*p* < 0.001 after Bonferroni corrections).

Further analyses demonstrate that eight of nine wild populations of ring‐tailed lemurs can be geographically grouped into two structured genetic clusters: a western cluster (BEZA, TNP) and an eastern cluster (ANJA, SAKA, TSARA, BER, AMB, and BLK), as indicated by the highest value of Δ*K* (Figure 2b). Cluster 1 comprised individuals from the six eastern localities and one central locality (61 of 106 individuals), and Cluster 2 comprised of individuals from the two western localities (45 of 106 individuals). The analysis was repeated with each of the *K* = 2 clusters separately following Evanno et al. (2005) and found that Cluster 1 (eastern localities) could be further subdivided into *K* = 2 geographically structured clusters (southern, subcluster 1: AMB, BLK, BER, and ISALO; northern, subcluster 2: ANJA, SAKA, and TSARA; Figure 2b). We found that Cluster 2 (western localities) was comprised of one genetic cluster, as the mean *L*(*K*) could not confidently exclude *K* = 1. Mean *L*(*K*) and Δ*K* plots of all Structure  runs are provided in supplementary information (Figure S1).

We detected similar patterns of structuring in the discriminant analysis of principal components (DAPC, Figure 2a) between the northern, southern, and western localities; however, according to the smallest BIC value, *K*‐means clustering estimated four genetic clusters. The majority of individuals clustered geographically with southern localities (AMB, BLK, BER) showing a higher membership probability to Cluster 1, northern localities (ANJA, SAKA, TSARA) to Cluster 2, and the central locality (ISALO) to Cluster 3. Individuals in the western localities (BEZA and TNP) showed higher membership probabilities to Cluster 4. From our initial analyses, we could not confidently group ISALO, as our Structure results indicated that ISALO clustered with southern localities (BER‐AMB‐BLK) despite the distance (301 km) and our DAPC results grouped the site into its own central cluster. Upon further investigation, we did find support in our Structure analysis for higher *K*‐values (*K* = 3–4, Figure S2), supporting the DAPC results and grouping ISALO into a separate central cluster.

**FIGURE 4:**
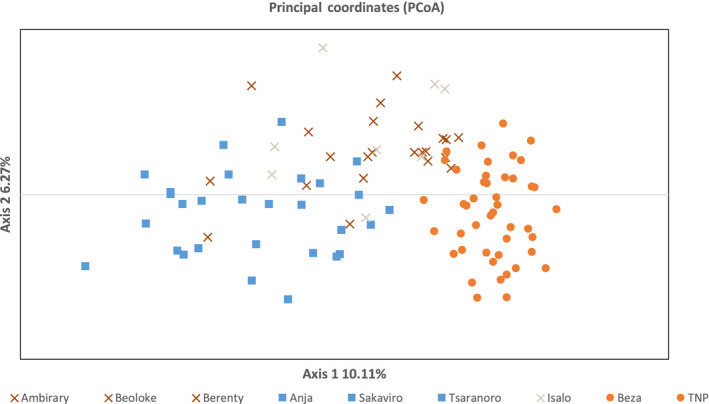
Principal coordinate analysis (PCoA). Data points are color coded according to DAPC analysis (orange: west; beige: central; blue: North; brown: South) while marker shapes correspond to STRUCTURE analysis (•: Western; □: North; x: South)

The principal coordinate analysis (PCoA; Figure 4) shows loose clustering between sampling localities according to geographic location. Western localities (BEZA and TNP) clustered together along axis one, while axis 2 separated sampling localities between north (ANJ, SAKA, TSARA) and south (AMB, BLK, BER). Though these results indicate geographic separation, the PCoA does show overlap between sampling localities in west, central, and southern Madagascar.

## 4 DISCUSSION

### 4.1 Genetic diversity and population structure

Although most of Madagascar's ring‐tailed lemur populations are geographically isolated, evidence described herein demonstrate that they have retained high levels of genetic diversity, with moderate genetic differentiation among populations despite being separated by a relatively large geographic distance. We describe levels of genetic diversity (MNA = 6.056, AR = 4.74, global *H*_O_ = 0.811; Table 3) that are higher than those found in any other strepsirrhine, including mouse lemurs (MNA = 2.75–4.38, *H*_O_ = 0.557–0.695; Olivieri, Sousa, Chikhi, & Radespiel, 2008; Radespiel, Rakotondravony, & Chikhi, 2008; Table 5). Though differing in size, habitat type, and protection status, the nine localities sampled in our study showed similar levels of genetic diversity. Interestingly, ISALO, the largest forest included in this analysis (81,500 ha), showed one of the lowest levels of allelic diversity (AR = 4.19) and some of the highest levels of heterozygosity (*H*_O_ = 0.85). In fact, the genetic diversity within ISALO was most similar to the smallest fragment included in our study, SAKA (14 ha, AR = 4.09, *H*_O_ = 0.86; Table 3). This is likely due to the relatively small number of markers used and our limited sample size (*n* = 8 individuals) at the time of this analysis.

**TABLE 5:** Indices of allelic diversity across twelve lemur taxa, including minimum, maximum, and mean number of alleles (MNA), observed (*H*_o_) and expected (*H*_e_) heterozygosity,  and population differentiation (*F*_ST_)

Family	Species	Sample	Marker	*n* ind.	*n* sites	MNA			*H*_o_	*H*_e_	*F*_ST_		
						Min	Max	Mean			Min	Max	Mean
Daubentoniidae	*Daubentonia madagascariensis* ^1^	Tissue	SNPs	12	8	–	–	–	–	–	(0.129)	(0.194)	(0.164 ± 0.033)
Cheirogaleidae	*Microcebus bongolavensis* ^2^	Tissue	8 microsats	45	3	3.63	5.00	4.17	0.557	0.582	0.057	0.102	0.076 ± 0.023
	*Microcebus danfossi* ^2^	Tissue	8 microsats	78	7	2.75	6.63	4.91	0.628	0.654	0.025	0.195	0.096 ± 0.049
	*Microcebus murinus* ^3^	Tissue	10 microsats	167	3	–	–	–	–	–	0.004	0.016	–
	*Microcebus ravelobensis* ^4^	Tissue	8 microsats	187	12	–	–	–	0.695	0.692	−0.002	0.122	0.052 ± 0.027
	*Microcebus ravelobensis* ^2^	Tissue	8 microsats	205	8	4.38	6.50	5.76	0.615	0.605	0.006	0.156	0.072 ± 0.035
Lepilemuridae	*Lepilemur edwardsi* ^5^	Tissue	14 microsats	20	2	3.86	4.00	–	–	–	−0.09	1.00	–
Lemuridae	*Eulemur cinereiceps* ^6^	Tissue	26 microsats	53	4	2.71*	3.36*	3.06*	0.520	0.527	0.020	0.076	0.054 ± 0.019
	*Varecia rubra* ^7^	Tissue	15 microsats	32	2	1.0	8.0	5.03	0.666	0.643	–	–	0.077
	*Varecia variegata* ^8^	Blood, feces	16 microsats	55	5	2.47*	3.23*	2.84*	–	–	0.039	0.291	0.197 ± 0.084
	*Varecia variegata* ^9^	Blood, feces	10 microsats	209	19	2.20	4.70	3.42	0.519	0.573	0.002	0.441	0.247 ± 0.094
	*Lemur catta* ^10^	Feces	6 microsats	30	3	4.83	5.83	5.27	0.797	0.703	0.05	0.11	–
	*Lemur catta* ^11^	Feces	6 microsats	106	9	4.33	8.67	6.06	0.811	0.775	0.034	0.183	0.129 ± 0.032
Indriidae	*Propithecus tattersali* ^12^	Feces	13 microsats	82	3	(3.43)*	(3.99)*	–	(0.707)	(0.621)	(0.136)	(0.160)	(0.147)
	*Propithecus tattersali* ^13^	Feces	13 microsats	224	9	3.00	6.00	4.92	0.690	0.660	0.010	0.300	0.119 ± 0.067
	*Propithecus verreauxi* ^14^	Tissue	7 microsats	77–131	10–28	–	–	–	–	–	0.024	0.075	0.052
	*Propithecus perrieri* ^15^	Feces	24 microsats	42	3	3.58	4.25	3.83	0.610	0.640	0.023	0.061	0.039
	*Indri indri* ^16^	Blood, tissue	17 microsats	43	3	4.81*	5.36*	–	–	0.850	0.0746	0.129	0.105

Note

*F*_ST_ comparisons are among sampling localities unless otherwise noted. Values in parentheses denote comparisons among inferred clusters. Asterisk values denote allelic richness of sampling localities.

^1^ Perry et al. (2013).

^2^ Olivieri et al. (2008).

^3^ Fredsted, Pertoldi, Schierup, and Kappeler (2005).

^4^ Radespiel et al. (2008).

^5^ Craul et al. (2009).

^6^ Brenneman et al. (2012).

^7^ Razakamaharavo, McGuire, Vasey, Louis, and Brenneman (2010).

^8^ Holmes et al. (2013).

^9^ Baden et al. (2014).

^10^ Clarke et al. (2015).

^11^ This study.

^12^ Quéméré, Louis, Louis, Ribéron, Chikhi, and Crouau‐Roy (2010).

^13^ Quéméré, Crouau‐Roy, Crouau‐Roy, Rabarivola, Louis, and Chikhi (2010).

^14^ Lawler, Richard, and Riley (2003).

^15^ Salmona et al. (2015).

^16^ Nunziata et al. (2016).

Nevertheless, despite the limited number of markers used in this study, we found continuous patterns of structure across this species' range, including subdivision of eastern localities into northern and southern groups. Though there are large geographic distances between localities, our *F*_ST_ results indicated only moderate differentiation between sites. This may be attributed to the dispersal ability of this species. Being the most terrestrial of living lemurs (Jolly, 1966; Sussman, 1972, 1974), ring‐tailed lemurs can disperse more easily across nonforested areas than forest‐dependent arboreal species. Furthermore, the largest river drainage systems in southern Madagascar are seasonal and therefore do not pose permanent dispersal barriers to this species; in fact, they may actually be used as dispersal corridors (Goodman et al., 2006).

There was strong evidence for isolation‐by‐distance (IBD), meaning there was a positive correlation between genetic and geographic distances among populations. There are currently no records of subfossil ring‐tailed lemurs outside of their current distribution (Godfrey, Jungers, Simons, Chatrath, & Rakotosamimanana, 1999), suggesting that this broad geographical range has been stable through geological time (Goodman et al., 2006). If localities had been isolated for a long time, we would expect genetic drift to erase any pattern of IBD (Baden et al., 2014; Olivieri et al., 2008). Therefore, there is potentially movement and relatively recent interconnectivity between ring‐tailed populations via river basins (e.g., Mandrare River; Goodman et al., 2006). Genetic structure can result from limited gene flow or from historical events such as fragmentation; however, distinguishing between these processes can be challenging, especially when demography is unknown and forest fragmentation is recent.

Measures of genetic diversity, gene flow, and population structure are subjected to time‐lag effects (Epps & Keyghobadi, 2015). For instance, *F*_ST_ values typically reflect historic rather than current population structure if populations have not yet reached migration–drift equilibrium (Whitlock & McCauley, 1999). Moreover, heterozygosity is slow to decline in previously large populations following a genetic health bottleneck (Cornuet & Luikart, 1996), a pattern which has been documented in the western localities of this species range (BEZA and TNP; Parga et al., 2012). Because deforestation has occurred within the last few decades (Brinkmann et al., 2014; Clarke et al., 2015; Gardner & Davies, 2014), it may therefore be too recent to gauge whether habitat loss has negatively impacted genetic diversity and gene flow in this species (Keyghobadi, Roland, Matter, & Strobeck, 2005; Nei, Maruyama, & Chakraborty, 1975).

### 4.2 Conservation implications

Genetic diversity is lost more rapidly within fragmented and isolated habitats, elevating a species' extinction risk (Frankham, 1995, 2003, 2005). Our results indicate that ring‐tailed lemur populations have high levels of genetic diversity. While this is encouraging for the conservation of the species, this may reflect past, not current population processes. Though this species is considered the most ecologically flexible lemur, exploiting anthropogenic landscapes and persisting in small fragments (Cameron & Gould, 2013; Gabriel, 2013; LaFleur & Gould, 2009; Sauther et al., 2006), they are significantly affected by fragmentation and occur at lower densities in poorer habitats (Eppley, Santini, Tinsman, & Donati, 2020; Gabriel, 2013; Kelley, 2011; Sussman et al., 2003). In addition, continued fragmentation and further isolation, coupled with climate change, may prove too much for this historically abundant lemur species. Climatic cycles have been shown to strongly affect mortality rate within this species; a 2‐year drought period in southwestern Madagascar resulted in a tenfold increase (3%–27%) in mortality among adult populations in this region (Gould et al., 2003).

Our future work aims to increase sampling efforts in underrepresented and unprotected regions, to reflect the full geographic range of this species and provide a species‐wide genetic health assessment (e.g., Calkins & Baden, in review). Moreover, this dataset forms the basis for future landscape genetics analyses which will be used to infer migration and gene flow across the species' remaining range. Because ring‐tailed lemurs are highly terrestrial and are suspected to utilize river basins as dispersal corridors (Goodman et al., 2006), they may have been able to disperse across Madagascar more easily than the more restricted arboreal lemur species. We can use landscape genetic analyses to test this hypothesis to not only better understand what facilitates and impedes gene flow, but also to develop targeted management plans moving forward.

## CONFLICT OF INTEREST

None declared.

## AUTHOR CONTRIBUTION

**Aparna Chandrashekar:** Data curation (lead); Formal analysis (lead); Investigation (equal); Visualization (lead); Writing‐original draft (lead); Writing‐review & editing (equal). **Jessica A. Knierim:** Data curation (equal); Formal analysis (equal); Funding acquisition (equal); Investigation (equal); Writing‐review & editing (equal). **Sohail Khan:** Formal analysis (supporting); Investigation (supporting); Writing‐review & editing (equal). **Dominique L. Raboin:** Formal analysis (supporting); Investigation (supporting); Writing‐review & editing (equal). **Sateesh Venkatesh:** Formal analysis (supporting); Investigation (supporting); Writing‐review & editing (equal). **Tara A. Clarke:** Data curation (equal); Formal analysis (supporting); Funding acquisition (equal); Investigation (equal); Resources (equal); Writing‐review & editing (equal). **Frank P. Cuozzo:** Formal analysis (supporting); Funding acquisition (equal); Investigation (equal); Writing‐review & editing (equal). **Marni LaFleur:** Data curation (equal); Funding acquisition (equal); Investigation (equal); Resources (equal); Writing‐review & editing (equal). **Richard R. Lawler:** Formal analysis (equal); Visualization (equal); Writing‐original draft (equal); Writing‐review & editing (equal). **Joyce A. Parga:** Data curation (equal); Formal analysis (supporting); Funding acquisition (equal); Investigation (equal); Resources (equal); Writing‐review & editing (equal). **Hantanirina R. Rasamimanana:** Formal analysis (supporting); Investigation (equal); Writing‐review & editing (equal). **Kim E. Reuter:** Formal analysis (supporting); Writing‐review & editing (equal). **Michelle L. Sauther:** Formal analysis (supporting); Funding acquisition (equal); Investigation (equal); Writing‐review & editing (equal). **Andrea L. Baden:** Conceptualization (lead); Data curation (equal); Formal analysis (equal); Funding acquisition (equal); Investigation (equal); Resources (equal); Supervision (lead); Visualization (equal); Writing‐original draft (lead); Writing‐review & editing (equal).

## Supporting information

Fig S1‐S2

Appendix S1

## Data Availability

Raw data are publicly available on Zenodo (https://doi.org/10.5281/zenodo.3750377, https://doi.org/10.5281/zenodo.3750382).
