# Supplementary material for: Genetic population structure of endangered ring‐tailed lemurs (Lemur catta) from nine sites in southern Madagascar
Source: Ecol Evol. 2020 Jul 16;10(15):8030–43. doi: 10.1002/ece3.6337 (PMC7417237; doi:10.1002/ece3.6337)
Supplement: Supplementary file 1 — Fig S1‐S2 [file ECE3-10-8030-s001.docx]

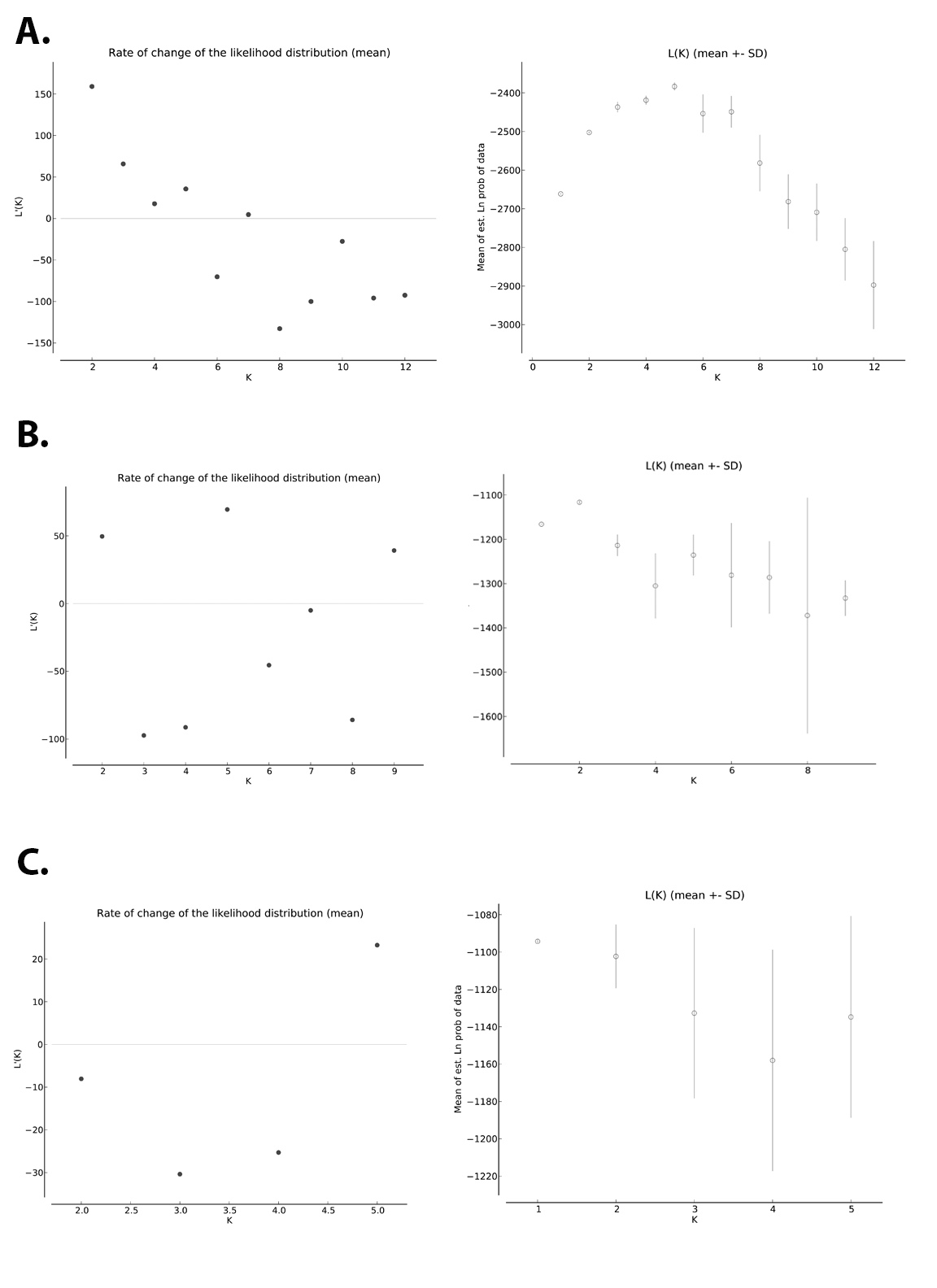
Supplementary Information

**Supplementary Figure S1.** STRUCTURE Analysis. Mean L(*K*) and ∆*K* of STRUCTURE analysis (A) of nine localities and 106 individuals. (B) of 7 eastern localities (ANJA, SAKA, TSARA, ISALO, AMB, BER, BLK) and 61 individuals. (C) 2 western localities a (BEZA and TNP) and 45 individuals.


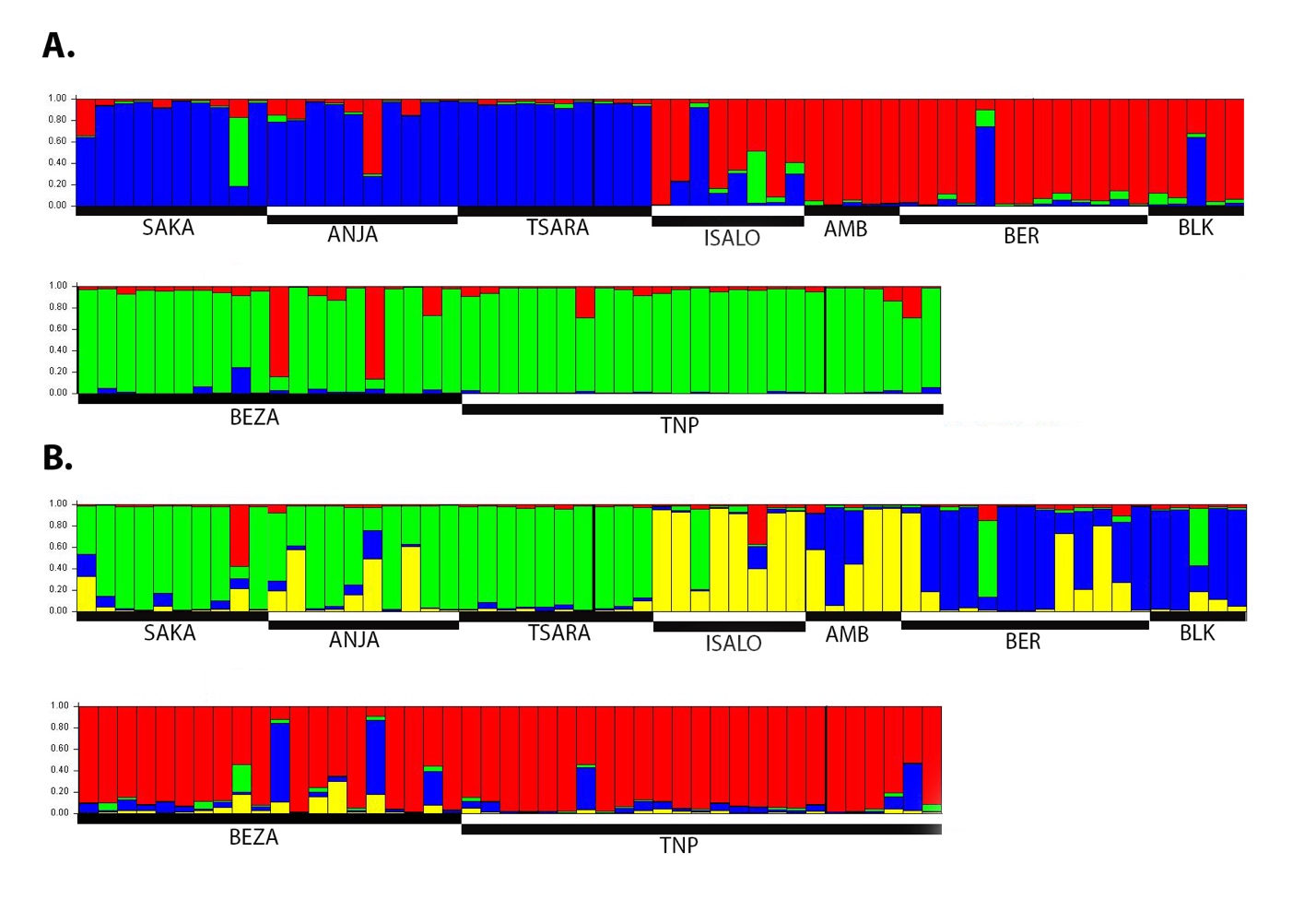


**Supplementary Figure S2.** STRUCTURE Analysis. Each bar illustrates the proportional membership (Q) of each individual lemur belonging to the genetic clusters K=3 (A) and K=4 (B).
