# Supplementary material for: Genetic population structure of endangered ring‐tailed lemurs (Lemur catta) from nine sites in southern Madagascar
Source: Ecol Evol. 2020 Jul 16;10(15):8030–43. doi: 10.1002/ece3.6337 (PMC7417237; doi:10.1002/ece3.6337)
Supplement: Supplementary file 2 — Appendix S1 [file ECE3-10-8030-s002.docx]

| Appendix**.** Primer size range, sequence, repeat motifs, annealing temperatures, and GenBank accession number of the six microsatellite markers used. References: (1) Merenlender, 1993; (2) Pastorini et al., 2005; (3) Zaonarivelo et al., 2007 | | | | | | | |
| --- | --- | --- | --- | --- | --- | --- | --- |
| Locus | Size Range (bp) | Sequence | Repeat Motif | Annealing Temp (°C) | GenBank accession no. | Reference |  |
| L-2 | 179-203 | F: atagacatccagagataagg R: ggcacctctagactctgtta | (GT)_9_(AG)_3_(GT)_4_ | 48 | ---- | 1 |  |
| Lc5 | 127-151 | F: tgggacaccttttgttatcag R: gatttctcaggacatccatag | (TC)_15_TT(TC)_6_TG(TC)_4_TG(TC)_6_ | 60 | AY366441 | 2 |  |
| Lc6 | 248-270 | F: ctttcctgttcttccactcag R: ccacagcctgcgattattgc | (TC) _14_AC(TC)_11_ | 60 | AY366442 | 2 |  |
| Lc7 | 172-198 | F: acctcccagcctattcacag R: ggagtggggacttgaatagc | (CA)_21_ | 60 | AY366443 | 2 |  |
| 69HDZ267 | 156-178 | F: acctccataacataagcacacg  R: agccagaataaagtcagggg | (CA)_19_ | 55 | EF093488 | 3 |  |
| 69HDZ299 | 238-262 | F: tgtctatgggtgggttttgtc  R: tagcattgtgattttctgttacatta | (CA)_16_ | 58 | EF093489 | 3 |  |
|  | | | | | | | |
